# Tigers in the Terai: Strong evidence for meta-population dynamics contributing to tiger recovery and conservation in the Terai Arc Landscape

**DOI:** 10.1371/journal.pone.0177548

**Published:** 2017-06-07

**Authors:** Kanchan Thapa, Eric Wikramanayake, Sabita Malla, Krishna Prasad Acharya, Babu Ram Lamichhane, Naresh Subedi, Chiranjivi Prasad Pokharel, Gokarna Jung Thapa, Maheshwar Dhakal, Ashish Bista, Jimmy Borah, Mudit Gupta, Kamlesh K. Maurya, Ghana Shyam Gurung, Shant Raj Jnawali, Narendra Man Babu Pradhan, Shiv Raj Bhata, Saroj Koirala, Dipankar Ghose, Joseph Vattakaven

**Affiliations:** 1 WWF Nepal, Baluwatar, Kathmandu, Nepal; 2 Department of Forest, Babarmahal, Kathmandu, Nepal; 3 National Trust for Nature Conservation, Lalitpur, Nepal; 4 Department of National Park and Wildlife Conservation, Babarmahal, Kathmandu, Nepal; 5 WWF India, New Delhi, India; 6 WWF-Tigers Alive, New Delhi, India; National Zoological Park, UNITED STATES

## Abstract

The source populations of tigers are mostly confined to protected areas, which are now becoming isolated. A landscape scale conservation strategy should strive to facilitate dispersal and survival of dispersing tigers by managing habitat corridors that enable tigers to traverse the matrix with minimal conflict. We present evidence for tiger dispersal along transboundary protected areas complexes in the Terai Arc Landscape, a priority tiger landscape in Nepal and India, by comparing camera trap data, and through population models applied to the long term camera trap data sets. The former showed that 11 individual tigers used the corridors that connected the transboundary protected areas. The estimated population growth rates using the minimum observed population size in two protected areas in Nepal, Bardia National Park and Suklaphanta National Park showed that the increases were higher than expected from growth rates due to in situ reproduction alone. These lines of evidence suggests that tigers are recolonizing Nepal’s protected areas from India, after a period of population decline, and that the tiger populations in the transboundary protected areas complexes may be maintained as meta-population. Our results demonstrate the importance of adopting a landscape-scale approach to tiger conservation, especially to improve population recovery and long term population persistence.

## Introduction

The tiger (*Panthera tigris*), arguably Asia’s most iconic large carnivore, has been extirpated from over 93% of its historic range [1]. In order to protect and recover the remaining populations scattered throughout the tiger range, conservationists have identified 20 landscapes with the greatest potential for conservation and long term persistence of wild populations [2]. Within these landscapes protected areas are now increasingly becoming insular as the matrix is converted to anthropogenic landuses. Tigers are especially vulnerable to such habitat loss and fragmentation [3–6] because a critical stage in tiger life history is dispersal of sub-adults from natal areas so they can establish territories and mate [7]. Dispersal of sub-adults from natal areas thus greatly influences tiger ecology, demography, and genetic variability.

There is mounting evidence of increasing tiger presence in human-dominated matrices [8]. But tigers that attempt to navigate a human dominated matrix can become fatal victims of human-tiger conflicts [7]. Alternatively, tigers that attempt to establish territories within the protected areas can come into conflict with established territorial animals, also with fatal consequences that disrupt reproduction, recruitment, and demographics for several generations [7]. Small, isolated tiger populations are also susceptible to inbreeding depression [9, 10]. Thus habitat connectivity is especially important for long-term tiger conservation [2, 11], and a tiger conservation strategy implemented at a landscape scale should strive to increase the chances of dispersal and survival of dispersing tigers by conserving habitat corridors that enable tigers to traverse the matrix with minimal conflict.

In the past, the expansive forest and Terai grassland-savannas along the base of the Himalayas supported high densities of tigers and prey [12]. Historical hunting records from Nepal dating back to 1938 describe Royal hunts that killed 120 tigers in 2 months from a small locality [13], suggesting immigrant tigers rapidly occupied habitats when residents were removed. However, the once contiguous tiger populations are now fragmented and isolated as sub-populations due to extensive habitat conversion. Smith *et al*. [14] identified three tiger sub-populations in the Nepal Terai, centered around three major protected areas, namely Suklaphanta National Park, Bardia National Park, and Chitwan National Park, with a few resident tigers in intervening forest patches, suggesting some remnants of historical dynamics. Genetic evidence indicates the presence of migrants in the core areas confirming there is still some dispersal mediated gene flow across the landscape that maintains genetic diversity [15]. The premise of metapopulation management in landscapes is that linking protected core areas embedded within the landscape with ecological corridors can maintain small populations as meta-populations to increase population persistence [2, 16, 17]. Thus, a severely depressed or extirpated population in a protected area can recover through immigration from another source population, if ecologically linked. The Terai Arc Landscape (here after referred as Terai Arc) was designed to connect the protected areas with habitat corridors for such tiger metapopulation management [18].

The Terai Arc, identified as a priority tiger conservation landscape, stretches along the base of the Himalayas from south-central Nepal to north-western India. It has remnants of the highly productive tall grasslands and savannas—known as the Terai,—and the Himalayan subtropical broadleaf forests. But the productivity of these alluvial grasslands has also resulted in extensive forest conversion for agriculture, with associated settlements and infrastructure development, isolating the protected areas. Accepting the importance of landscape level conservation to recover tiger populations, the Government of Nepal endorsed a conservation plan for the Terai Arc [19].

**Fig 1:**
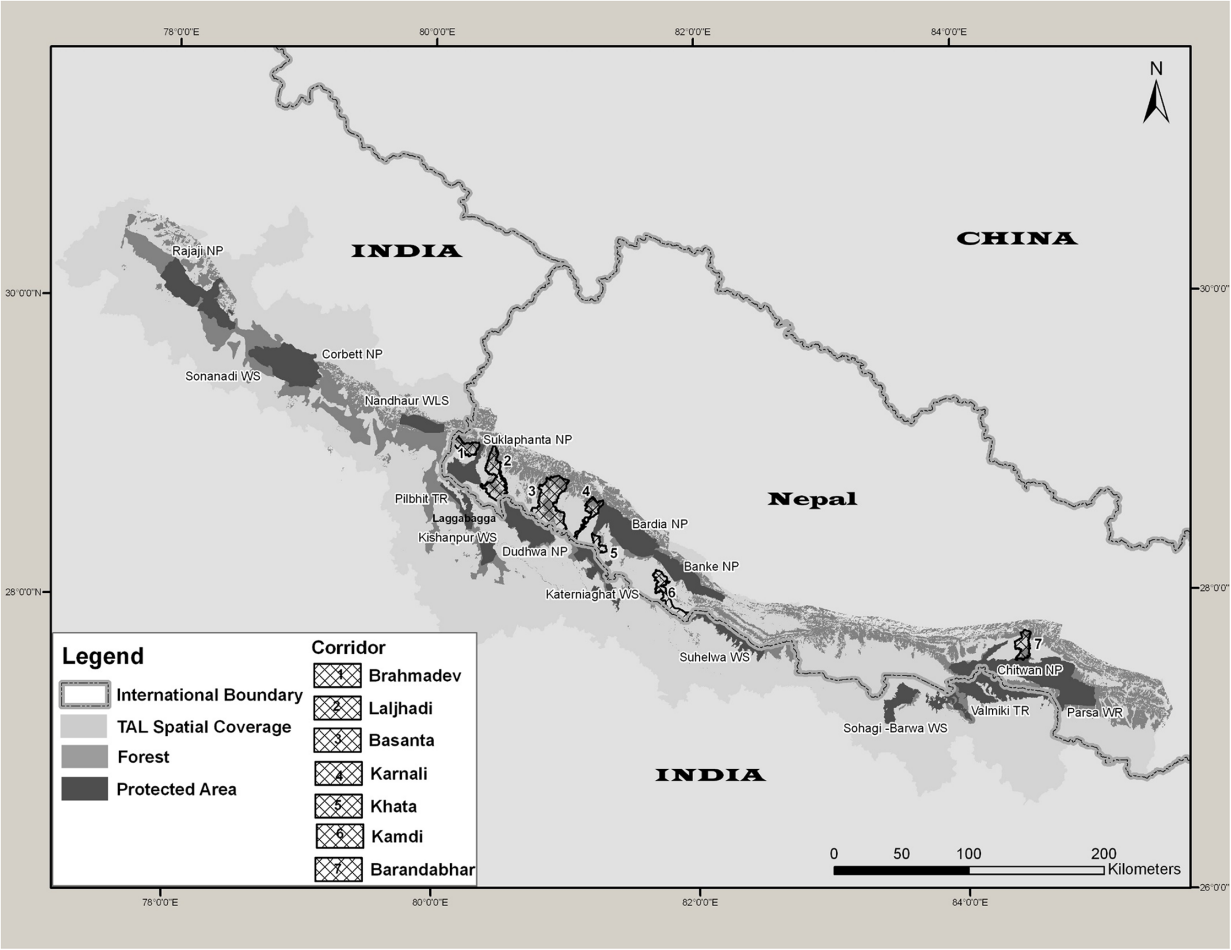
Terai Arc Landscape showing network of 16 protected areas in Nepal (5) and India (11) and forest corridor identified within the Nepal side of the landscape.

When the Terai Arc program was initiated in 2001, a landscape analysis identified several potential corridors that could provide ecological connectivity among the protected areas [18]. These included transboundary corridors between protected areas in Nepal and India (Fig 1). The analysis also highlighted several corridors that had bottlenecks and were in danger of becoming severed, and these corridors were prioritized for restoration for meta-population [20]. In the early and mid-2000’s poaching levels in Nepal’s protected areas rose steeply due to the period of civil unrest, which constrained park protection [21]. During this time, the estimated tiger populations in Bardia National Park and Suklaphanta National Park declined from 42 and 17 to 18 and 5, respectively [22, 23]. But in 2006 protection was restored and actions were taken to eliminate poaching [21]. Steps were also taken to restore and manage the corridors [24].

Here, we present evidence to show that these habitat corridors have enabled tiger recovery in Nepal’s protected areas and support the meta-population dynamics of tigers in transboundary protected areas complexes in the Terai Arc.

## Material and methods

### Study area

The Terai Arc covers an area of 50,911 km^2^ (Nepal: 24,710 km^2^, India: 26,201 km^2^) and stretches across 700 km in India and Nepal (Fig 1). The landscape contains almost all the forests of the Siwalik hills in the outer Himalayan range and the Terai regions of north-western India, and over 75% of the remaining forests of the Churia range of hills and Terai regions in south-western Nepal. All of the 16 protected areas of Nepal and India embedded within the landscape contain tiger populations of varying population densities, ranging from 0.65 to 16 tigers per 100 km^2^ [25].

There are three transboundary protected areas complexes in the landscape (Fig 1). These are: 1) the Chitwan National Park (CNP)-Parsa Wildlife Reserve (PWR) complex in Nepal that is linked with India’s Valmiki Tiger Reserve (VTR) through forests in the Churia range; 2) the Bardia-Banke National Park complex in Nepal that is connected with India’s Katerniaghat Wildlife Sanctuary (KWS) through the Khata corridor, and to Suhelwa Wildlife Sanctuary through the Kamdi corridor; and 3) Nepal’s Suklaphanta National Park (SuNP, 305 km^2^), which is linked with India’s Pilibhit Tiger Reserve-Kishanpur Wildlife Reserve complex through the Lagga Bagga forest. India’s Dudhwa Tiger Reserve (DTR) also has tenuous connectivity with SuNP.

### Methods

**Fig 2:**
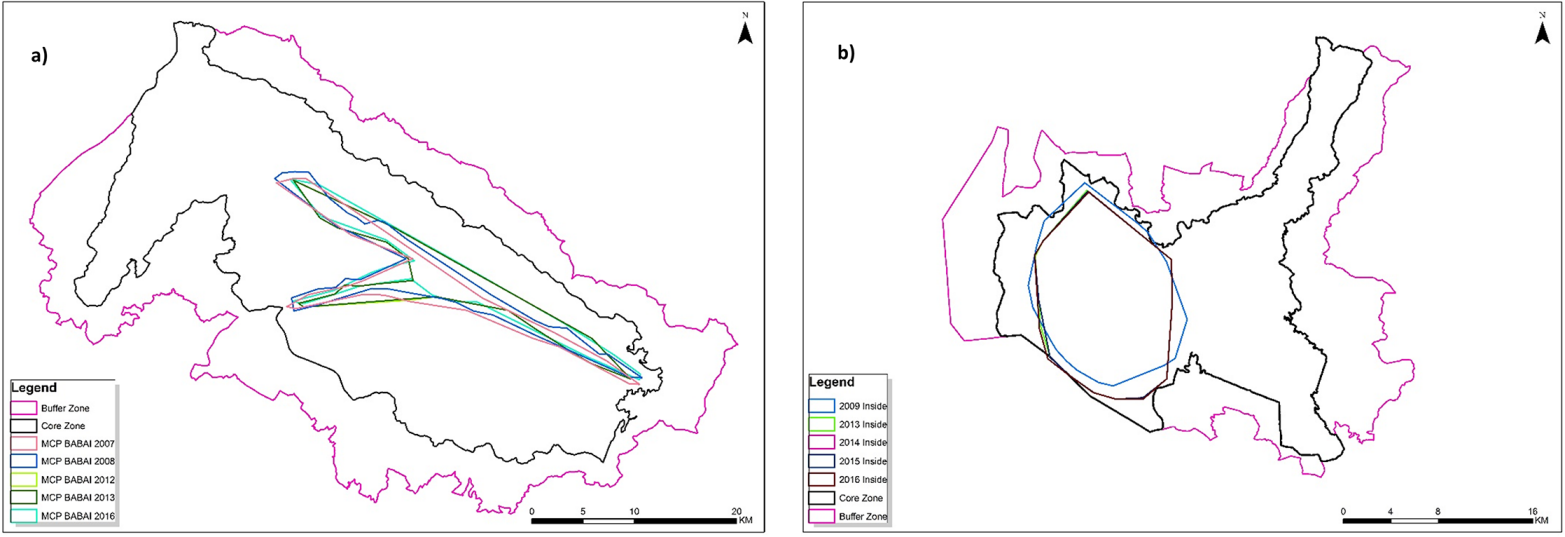
Minimum Convex Polygon (MCP) from multiyear camera trap surveys carried out in a) core area of Suklaphanta National Park; b) Babai valley within Bardia National Park.

We used the published data sets from multiple camera trap surveys carried out in the Terai Arc’s protected areas and corridors in Nepal and India between 2012 and 2016 [25–28], and 2011 survey carried out in the Churia range of CNP [29]. We also used the photographic capture datasets from the multiple camera trap surveys (n = 5 primary session) conducted in the winter season between 2009 and 2014 in SuNP and in Babai Valley (n = 5 primary session) of Bardia National Park (BNP) between 2005 and 2009. We followed standard camera trapping protocol [30]; thus, the camera trap field design does not vary between successive primary sessions (Table 1, Fig 2). Within core area, measuring 133.36 km^2^ (SD 1.15 km^2^) and 81.91 km^2^ (3.44 km^2^) for SuNP and BNP respectively, camera were placed in the grid (2 km X 2 km) formation at 1–1.5 km spacing for 15 days.

**Table 1:** Configuration of camera trap survey conducted in the Suklaphanta National Park (Core) and Babai Valley of Bardia National Park showing year of survey, minimum population size (M_t+1_), area surveyed (MCP; 100%) and number of camera trap deployed. Spacing between camera trap stations were set between 1.5–2 km and primary sampling occasion was set at 15 days period.

Protected Areas	Year Surveyed	Minimum Population Size (M_t+1_)	Area Surveyed (In km^2^)	Number of Camera trap location
SuNP				
	2009–2010	5	135	46
	2010–2011	8	N/A	46
	2011–2012	14	N/A	46
	2012–2013	14	132	46
	2013–2014	13	133	45
BNP (Babai Valley)				
	2006–2007	6	76	53
	2008–2009	4	83	62
	2011–2012	13	85	40
	2012–2013	14	84	40
	2015–2016	15	81	47

Individual tigers (sub-adults and adults) were positively identified from camera trap photographs from Nepal and India using Extract Compare software [31] and subsequently verified by experienced wildlife technicians. The locations of individuals were mapped using ArcGIS (ESRI, Ver 10.1) to show their capture locations. We also used capture locations of individuals that were found in corridors and protected areas to estimate a minimum area occupied by an individual (in km^2^) using Minimum Convex Polygons (MCP) [32]. The maximum Euclidean distances (in km) between individuals’ capture locations were also determined in ArcGIS. We used the observed population (minimum number, M_t+1_) size across multiple surveys to calculate the rates of change in tiger subpopulations in Nepal’s protected areas during the specified period using the conventional exponential population growth model [33, 34]: N_t+1_ = N_t_ + N_t_x R, where N_t_ is population size at time t, N_t+1_ is population size at time t+1, and R is the annual growth rate. Karanth et al. [33] estimated a 3% annual growth rate for tiger populations in Nagarhole National Park, India, and Miquelle et al. [34] proposed annual growth rates between 3–5%, with initial growth rates of 10% for Amur tigers. We therefore used these annual growth rates (3% and 10%) to calculate the expected population increase in SuNP using the population estimate from 2008, when the tiger population in SuNP had been severely depressed due to intense poaching [35]. We then calculated the annual growth rate in the tiger population in SuNP based on observed tiger numbers since 2008. The data were plotted, and polynomial curves were fit in Microsoft Excel.

## Results

### Tiger movements from camera trap data

**Fig 3:**
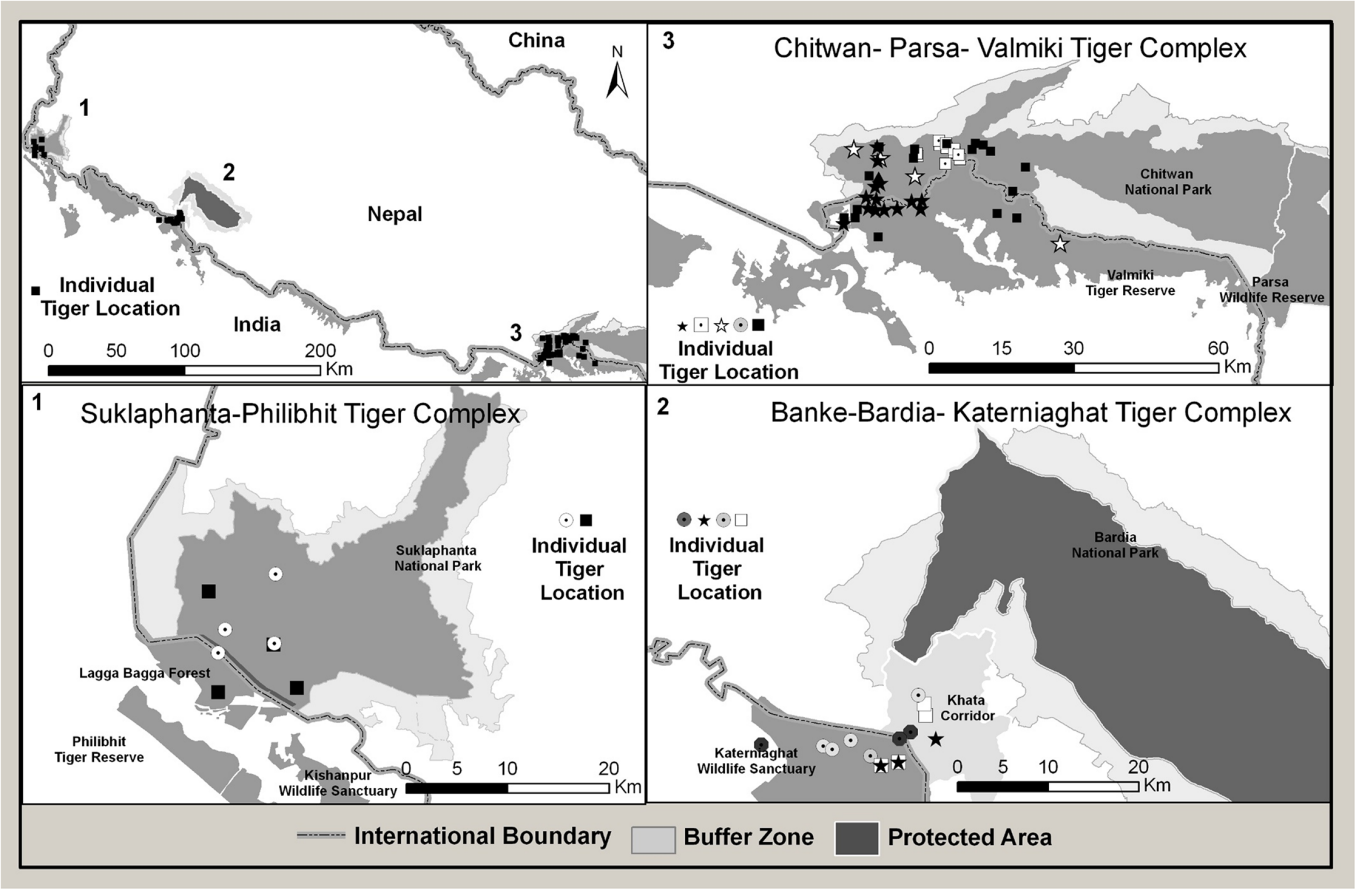
Spatial location of common tigers (n = 11) between transboundary protected areas in Terai Arc Landscape. Two tigers (2 male) common between Suklaphanta-Philibhit Tiger Complex, four tigers (3 male: 1 female) common between Banke-Bardia-Katerniaghat Tiger Complex and five tiger (4 male:1 female) common between Chitwan-Parsa-Valmiki Tiger Complex.

Over intensive efforts of 38,319 trap days covering 9111.78 km^2^ of tiger habitat showed eleven individual tigers were photographed by camera traps crossing the transboundary complexes in Nepal and India (Table 1). Of these, five (4 males, 1 females) were from the Chitwan-Parsa-Valmiki complex, four (3 males, 1 female) from the Bardia-Katerniaghat complex, and two (males) in the Suklaphanta-Lagga Bagga-Pilibhit complex (Fig 3). In the Chitwan-Parsa-Valmiki complex, one male that was detected in the eastern part of VTR, India was also photographed 43 km away in the western part of CNP, Nepal (Fig 3). A female tiger was photographed in both VTR and CNP at distances of over 17 km apart and the area included within the 100% MCP was over 113 km^2^. Another male photographed in both VTR and CNP, had furthest capture points of over 35 km, and a 100% MCP of over 248 km^2^. Another male was photographed in VTR, close to the border of CNP, and had a camera trap 100% MCP of 21 km^2^.

**Fig 4:**
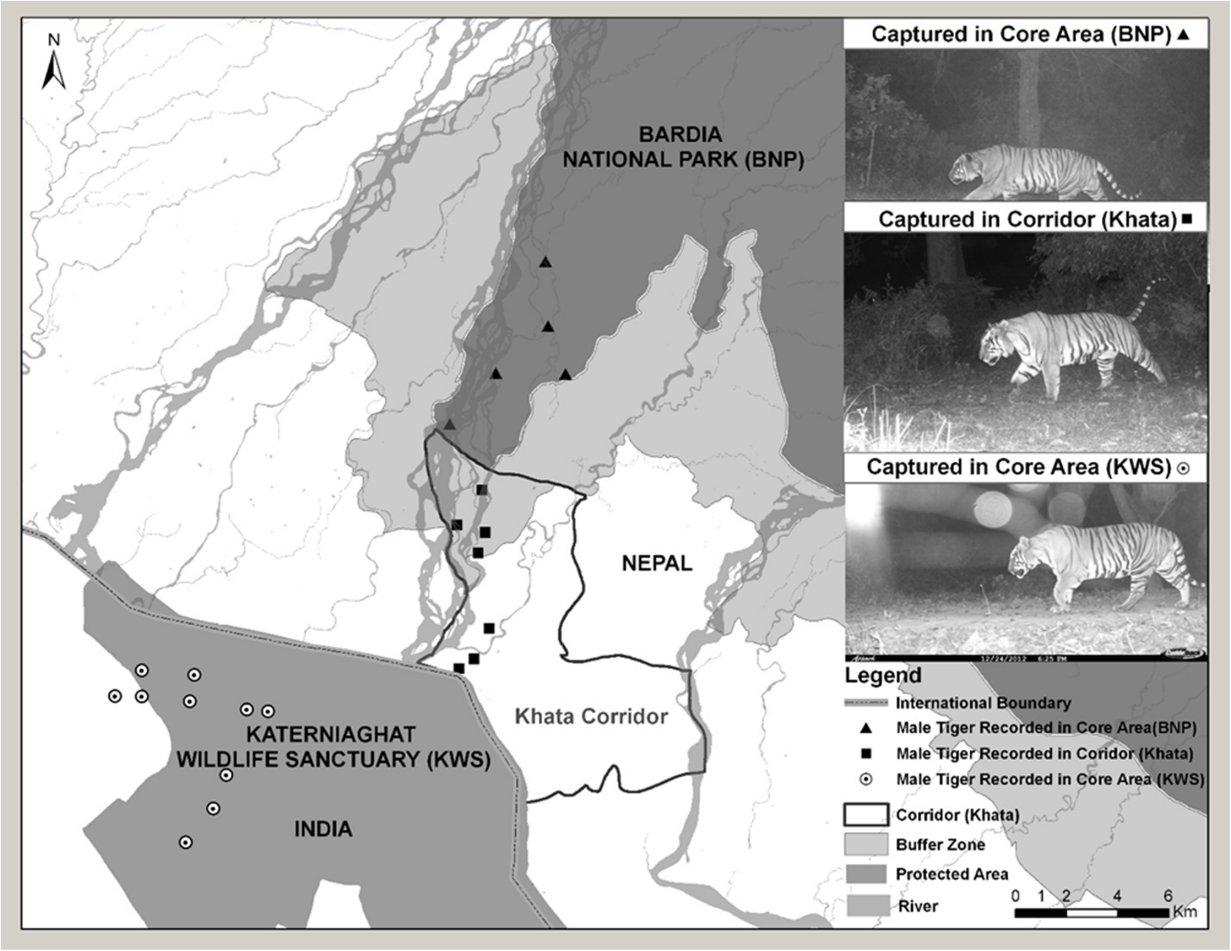
A male tiger captured along the Bardia-Katerniaghat forest matrix connected through Khata corridor forest in the western part of Terai Arc Landscape.

In the Bardia-Katerniaghat complex, two males were camera-trapped in Katerniaghat Wildlife Sanctuary (KWS), India, and in the buffer zone of Nepal’s BNP (Figs 3 and 4). Both animals were also photographed in the Khata corridor, which links the two protected areas. One of these males was camera trapped in KWS, Khata, and in BNP during surveys conducted between 2013 and 2016. A third male and a female were photographed in KWS and in the Khata corridor. Both animals photographed from the Lagga Bagga-SuNP were males, and each was captured >10 km (Fig 3). The 100% MCP for both animals were over 30 km^2^.

### Population trends

**Fig 5:**
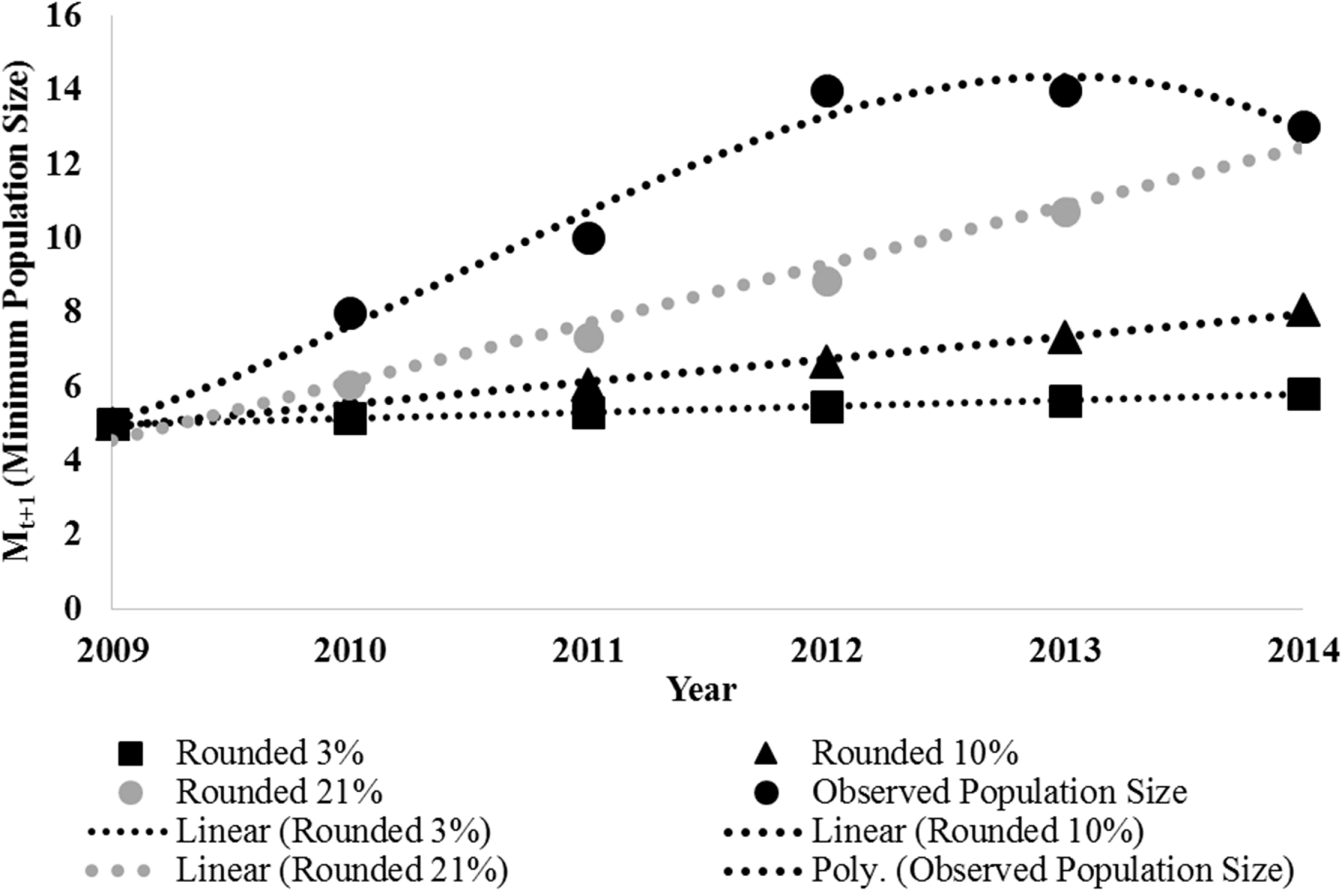
Fitted polynomial growth curve based on 3%, 10%, 21% growth rates regressed on minimum population size (M_t+1_) of camera trap tiger population surveyed in between 2009 and 2014 in core area of Suklaphanta National Park.

**Fig 6:**
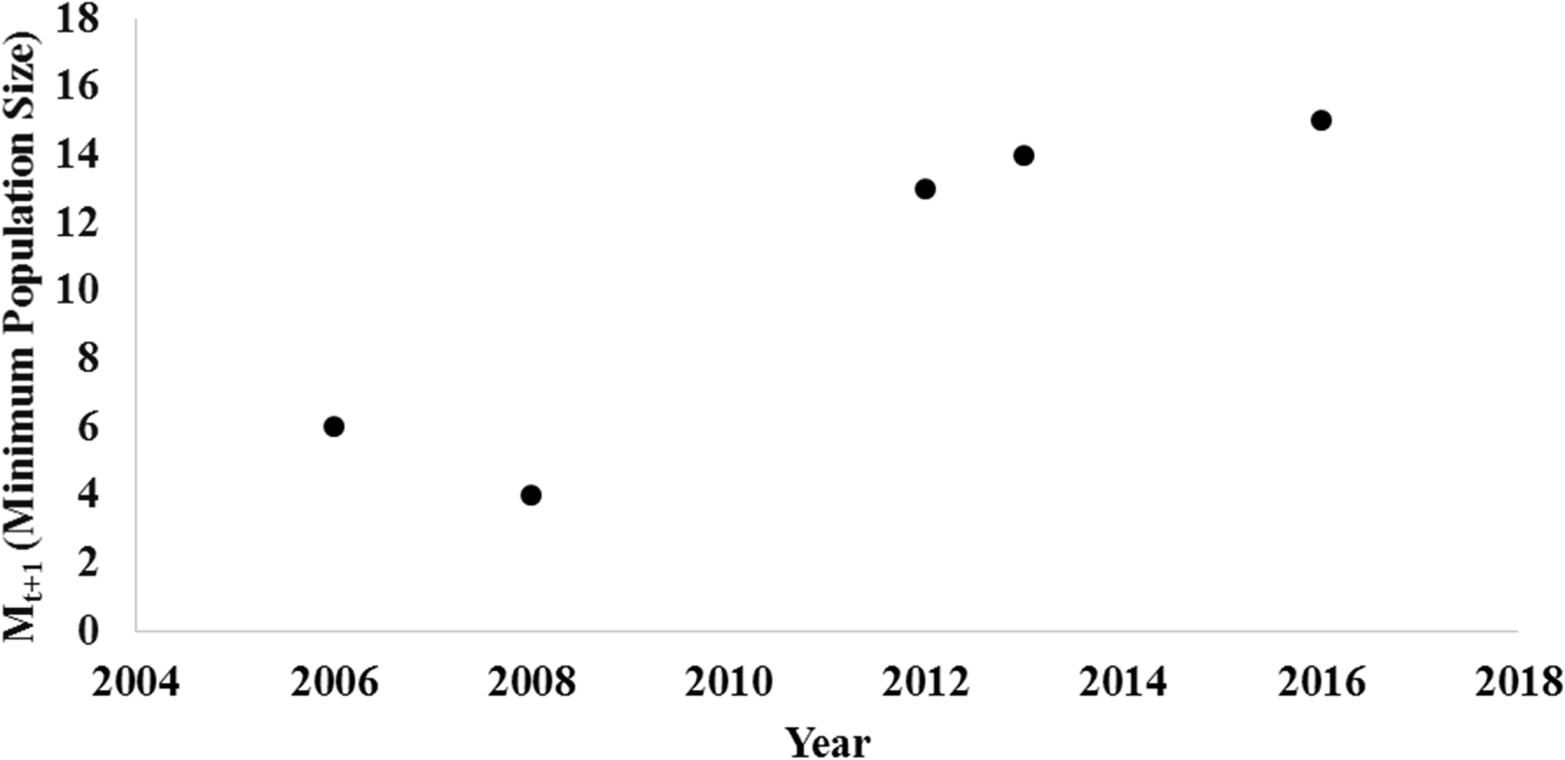
Trend in minimum population size (M_t+1_) of the tiger population based on adhoc camera trap tiger population surveyed in between 2006 and 2016 in the core area of Babai Valley within Bardia National Park.

The estimated population growth rates based on 3% and 10% increases from *in-situ* reproduction were not large enough to achieve the observed population from a low of 5 tigers in 2009 to 13 tigers in 2013 in SuNP (Table 1) and from a low of 6 tigers in 2006 to 14 tigers in 2014 in BNP’s Babai Valley. Achieving these observed populations would have required a 21% increase in SuNP (Fig 5) and 15% increase in Babai Valley. In SuNP, the initial observed population increase since 2008 was greater than expected from an annual increase of 21%, but the slowed down in 2013 (Fig 5). In Babai valley, population trend increased, and then stabilized after 2013 (Fig 6).

## Discussion

This study, based on the Government of Nepal’s long term tiger monitoring program, presents strong evidence to show recovery of tiger sub-populations (in BNP and SuNP) in Nepal’s protected areas in the Terai Arc after previous precipitous declines. We used the minimum number of recorded individuals (M_t+1_) derived from systematic camera trap survey designs that are uniform in sample area coverage, locations, duration and season over the monitoring period, 2006 to 2016, to compare the observed minimum population size (M_t+1_). The consistent and standard methodology allows comparison of data from multi-year surveys. Utility of the capture and recapture sampling framework is widely available with non-spatial open-model CR analyses for assessing the estimates (survival rates, recruitment) of tiger dynamics as per the studies done in India [36] and Thailand [37], however due to low sample size we used the conventional analytical techniques for the study.

### Tiger dispersal in the Terai

About five years after restoration of the corridor bottlenecks began in 2001 tiger presence was confirmed in two corridors; Khata and Basanta. More recent surveys of tiger habitat in the landscape in adjoining India indicated high tiger occupancy in transboundary corridors that suggested tigers in the landscape may be sustained through meta-population dynamics [38, 39]. But there was no empirical evidence to indicate that tigers may be dispersing between core areas.

Our camera trap surveys now confirm that at least 11 individual tigers are using the transboundary corridors that connect with Nepal and India’s protected areas (Fig 2). While detection of individual tigers in both protected areas and connecting corridors does not confirm dispersal, the supporting evidence based on spatial areas occupied and distances covered suggest these individuals were not resident tigers with territories that overlapped across protected area and corridor boundaries, but were transient, non-resident tigers.

The average territory size of female tigers in the Terai alluvial grassland-savannas, calculated using MCP analysis, is approximately 20 km^2^ [22]. But the area within a MCP calculated here for the 10 tigers photographed from both Nepal and India ranged from 21 to 248 km^2^. Although the numbers of detections for each tiger is low, the spatial areas occupied by these animals are much larger than the expected territory sizes of tigers in the Terai. There were also no recaptures in the same cameras or from adjacent cameras suggesting that these individuals did not patrol an established territory. The distances between camera traps in which tiger were photographed in our study are also consistent with distances travelled by dispersing tigers in the Nepal Terai, where a radio telemetry study in CNP showed that, on average, males dispersed 33 km and females 9.7 km from natal areas [7]. The earlier dates of capture in India’s protected areas indicate that they were moving from India into Nepal (Fig 3). Thus, the circumstantial evidence indicates that these transboundary tigers were non-territorial individuals that may have been dispersing from India’s protected areas through the transboundary corridors. Radio telemetric studies would provide definitive direct evidence of such dispersal behavior, and is highly recommended.

### Population growth rates in protected areas

The demographic data on tigers in Nepal’s protected areas since the period of decline in the mid-2000’s provide additional support data to indicate that population increases observed cannot be attributed to natural reproduction within the respective protected areas alone. Our analysis shows that the subsequent tiger recovery rate in SuNP was 21%, increasing from the low of 5 animals in 2009 to 13 in 2014 (Table 1, Fig 4). This growth rate is much higher than expected from 3% to 10% growth rates observed in tiger populations in India and the Russian Far East [33, 34].

The 2011–12 survey estimated the tiger population in Babai Valley at 13 animals (3 males and 10 females); an increase from 4 animals (2 males and 2 females) within just four years, which cannot be attributed to *in-situ* natural reproduction. Tigers photographed in the Khata corridor, adjacent to the buffer zone of BNP were also observed at the entrance to Babai Valley where it opens out to the Karnali River floodplain in the southern buffer zone (Fig 2). Therefore, immigration of tigers from India’s KWS along the Khata corridor and Karnali River flood plain into the Babai valley is possible, and would be a more parsimonious explanation for the high rates of tiger recovery in BNP. Satellite telemetry studies conducted in Nepal on rhinoceros in the Khata corridor shows regular movement of rhinoceros into KWS along the Khata corridor indicating its functionality as a wildlife corridor (DNPWC unpublished data).

### Metapopulation structure and population resilience of tigers in landscapes

Here, we present evidence to show that metapopulation dynamics may be contributing to tiger population recovery from near-extirpation levels in some protected areas of the Terai Arc, likely increasing tiger population resilience and persistence in the landscape. The observations present additional evidence confirming the importance of landscape-scale approaches to mega-vertebrate conservation. When the Terai Arc’s corridor conservation and restoration program was initiated in 2001, the transboundary corridors were identified as bottlenecks and prioritized for restoration. Without conservation interventions the habitat in the corridors would most certainly have been cleared, severing connectivity, which may not have allowed the tiger populations in Nepal to recover from the severe poaching events of the mid-2000s.

An example is the Basanta forest corridor that extends from the Churia mountain range in Nepal to DTR in India (Fig 1). The Basanta forest corridor was identified as a priority for conservation but did not receive early attention despite being flagged as a priority. The forest is now heavily settled by people and the forests are highly fragmented. Tiger presence has not been confirmed during our camera trap surveys, even though tiger presence was reported from parts of Basanta during the early 2000s. Furthermore, surveys in the far western regions of the Terai Arc, where it extends into northwestern India, have revealed that severed corridors have prevented colonization of suitable habitat in a protected area and several reserve forests, causing the tiger populations to be significantly depressed [40].

Analysis of trends in population dynamics in other tiger landscapes also support evidence for metapopulation structure of tigers in landscapes. Long term tiger monitoring in India’s Nagarhole National Park has shown that the population has fluctuated from 7.3 to 21.7 tigers/100 km^2^, with frequent turnover and arrival of tigers into the park [33]. Wikramanayake et al [2] have posited that this protected area is embedded within a large landscape spread across the Nilgiri mountain range in the Western Ghats, with habitat linkages that connect 12 protected areas. Thus, the observed dynamics suggests that metapopulation dynamics may be contributing to the persistence of the tiger population in Nagarhole, rather than only in-situ reproduction and recruitment.

Wikramanayake et al [2] also suggest similar dynamics in the Russian Far East, where tigers were almost extirpated in the 1940’s due to heavy hunting, but survived in adjacent forests in northeastern China [41]. Subsequently, when hunting was controlled in the Russian Far East tiger dispersal from China contributed to population recovery [41]. In an ironic twist, during the 1990’s, poaching in China’s northeastern region extirpated its tigers, but they are now being reestablished through dispersal from the population in the Russian Far East [42].

In a third example, evidence from Thailand’s Huai Kha Khaeng National Park, embedded within the large Western Forest Complex landscape in the Tenasserim mountain range, has shown that tiger numbers almost doubled to 56 animals from 2010 to 2012, with the addition of 24 tigers [37]. This recovery happened after improved patrolling in the protected area to curb intense poaching that had severely depressed the tiger population in the park. Such a near-doubling of tigers within 2–3 years can only be achieved through immigration, and not by in-situ reproduction alone. Thus, landscape scale analyses are providing more support for tiger movement between protected areas that contribute to population recovery and persistence in these core areas.

Studies of tiger genetics also provide supporting evidence for metapopulation structure of tigers in landscapes. In central India’s 45,000 km^2^ Satpura-Maikal landscape, tigers in protected areas that are linked by habitat corridors exhibited less genetic subdivision than tigers in protected areas that were isolated due to habitat loss over the past 150 years [17, 43]. Despite the anthropogenic habitat fragmentation in this landscape, some dispersal is still occurring between protected areas, and securing these corridors for conservation is considered a priority to ensure tiger persistence [16].

### Landscapes for metapopulation management and persistence of mega-vertebrates

Metapopulation management for conservation of wildlife evolved as a strategy when it become evident that populations in isolated protected areas embedded within landscape matrices of anthropogenic land use are vulnerable, but that linked subpopulations can increase overall population viability and persistence through dynamics of dispersal and colonization [44–48]. The population dynamics and tiger dispersal patterns in the Terai Arc supports our results, and demonstrate the importance of adopting a landscape-scale approach to tiger conservation, especially to improve population recovery. Studies of other large carnivores—lions (*Panthera leo*) [49] mountain lions (*Puma concolor*) [50, 51], jaguars (*Panthera onca*) [52], leopards (*Panthera pardus*)[53, 54], snow leopards (*Panthera uncia*) [55], bears (*Ursus* spp.)[56], wolves (*Canis lupus*) [57]—confirm the need for connected landscapes as a long term conservation strategy.

**Fig 7:**
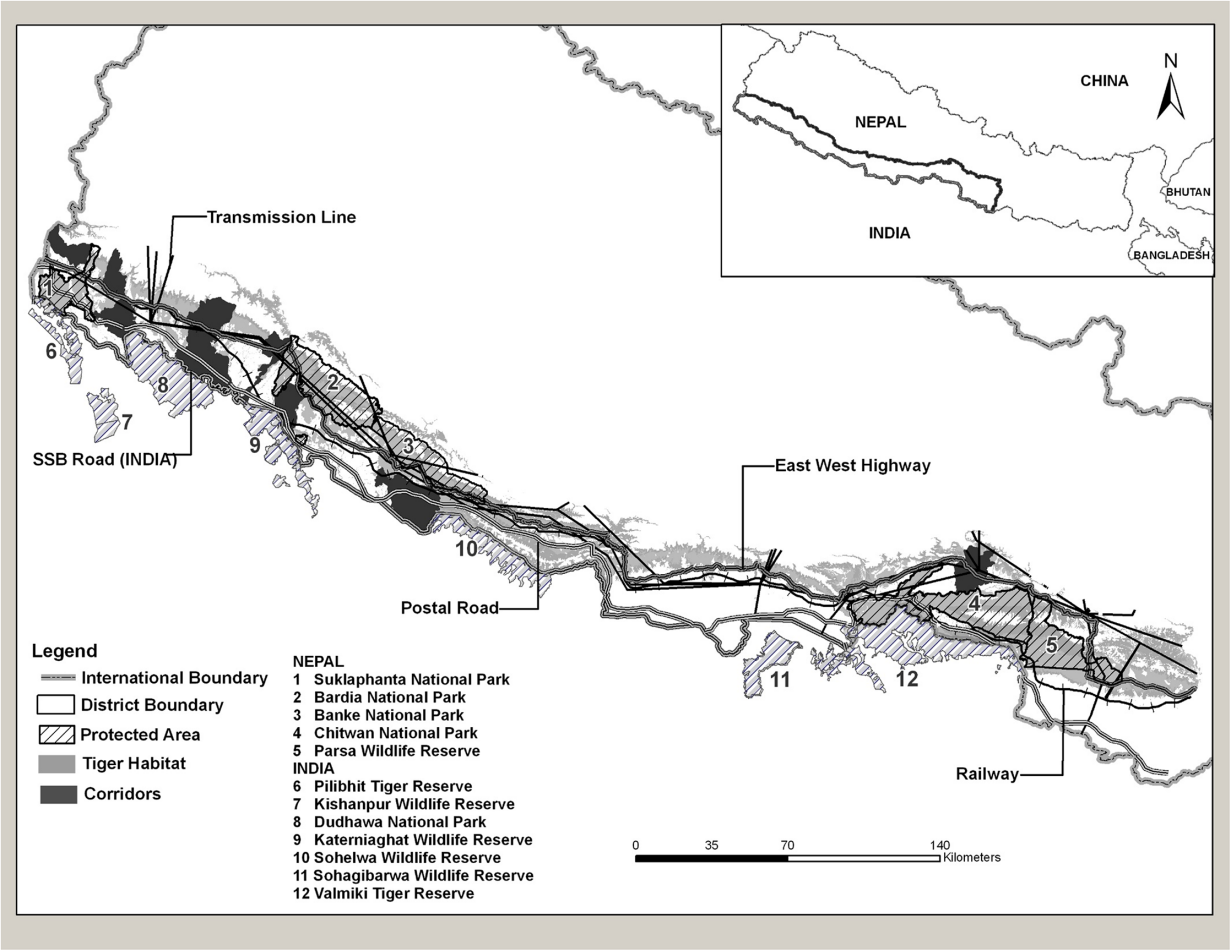
Infrastructure development (railways, highways and postal roads) along the transboundary tiger habitat between Nepal and India across Terai Arc Landscape.

But, conservation at landscape scales is a more challenging endeavor than a focus on specific sites because of the need to address conflicting land uses and land demands in the landscape and the processes to reconcile these conflicts [53, 58–61]. Negotiations with development agencies have been necessary in the Terai Arc. Two parallel roads being built on either side of the international border threaten to sever the transboundary corridors, and another road will pass through the Barandabhar corridor to the north of CNP in Terai Arc (Fig 7). Negotiations with governments in both India and Nepal have been necessary to integrate viaducts to allow wildlife movement, and maintain corridor functionality. In India, protection of wildlife corridors has been mandated by the Supreme Court [40], but such laws are still lacking in Nepal, which makes the task more difficult. This is likely true for many other countries. The increase in landscape-scale conservation initiatives worldwide for large vertebrates [49, 50, 56, 62] and the integration of wildlife corridors into development plans across the world [60, 63, 64] indicate that conservationists are now up to these challenges.

Large carnivores will remain conservation dependent species in a world where natural habitats will face increasing threats from development. Protected areas will continue to remain the cornerstones in a conservation strategy, but there is now enough compelling evidence to affirm that landscape connectivity is also important to ensure long-term persistence of these species [52–54, 64–66].
